# Management of early childhood caries through parental diet counselling: A pilot study in Bhopal, Madhya Pradesh, India

**DOI:** 10.6026/973206300222310

**Published:** 2026-04-30

**Authors:** Bharti Peshwani, Pankaj Goel, Sana Anwar, Anindo Majumdar, Ashish Khandelwal, Sangeeta Teleti, Shweta Kumari, Taiba Shaikh

**Affiliations:** 1 Department of Dentistry, All India Institute of Medical Sciences, Bhopal, Madhya Pradesh, India; 2 Department of Community and Family Medicine, All India Institute of Medical Sciences, Bhopal, Madhya Pradesh, India

**Keywords:** Diet counselling (DC), diet diary, dental caries, DMFT index, dietary habits, caries prevention

## Abstract

Untreated early childhood caries (ECC) is a significant global public health condition associated with pain, infection, sleeps disturbances and impaired psychosocial development. Therefore, it is of interest to evaluate the effectiveness of parental dietary counselling in managing Early Childhood Caries (ECC) among 39 children (≤6 years) at a tertiary care hospital in Bhopal. Diet was assessed using the Starting the Conversation (STC) questionnaire and a 24-hour diet diary, showing reduction after one year. There was a significant decrease in STC scores, sugar exposure and number of decayed teeth (p < 0.0001), with birth order exposure influencing liquid sugar exposure. Thus, parental dietary counselling effectively reduces sugar exposure and caries progression, supporting its role in ECC prevention and management.

## Background:

Early childhood caries (ECC) is defined as the presence of one or more decayed (non-cavitated or cavitated), missing (due to caries), or filled tooth surfaces in any primary tooth in a child up to 71 months of age or younger [1, 2]. Caries is the most common chronic disease of children, occurring five times more often than asthma and is seven times more common than hay fever [3, 4]. Caries-risk assessment needs to be done as early as possible before the commencement of disease to achieve the best management and outcomes for achieving a good oral health [5]. The factors responsible for ECC consist of vulnerable host, fermentable carbohydrate diet, presence of dental plaque and a high number of cariogenic microorganisms such as Streptococcus Mutans, Lactobacillus and most importantly time [6, 7]. Other risk factors include socio-economic status, working status of mother, oral hygiene habits and consumption of medications [8, 9]. The frequent intake of fermentable carbohydrate, especially sugar, has an important role in development of dental caries [10, 11]. Dietary counselling (DC) is a process where health professionals provide special training by individualized nutritional care to encourage people to make healthy food choices and healthy eating behaviours for caries prevention and management [12]. Starting The Conversation (STC) is a simplified screening instrument designed for assessing the patient's diet and helps in guiding nutritional counselling [13]. This tool was created by the Centre for Health Promotion and Disease Prevention at the University of North Carolina at Chapel Hill (USA) [14]. The eight-item dietary assessment focuses on dietary patterns that impact the development of chronic diseases. It is available in English and Spanish languages [15]. This tool identified healthful and unhealthful dietary behaviours in a diverse population highlighting its applicability in public health and primary care settings [16]. Hence STC is a relatively simple, valid and efficient tool for dietary assessment and intervention in the clinical setting [17]. Therefore, it is of interest to describe the role of parental dietary counselling among children with early childhood caries using the STC as dietary assessment tool.

## Methodology:

This study was conducted in the Department of Dentistry, All India Institute of Medical Sciences (AIIMS), Bhopal over a period of 1 year. The study population comprised of children with Early Childhood Caries (ECC) and their primary caregivers reporting to the Dental OPD. A Sample size of 39 children was selected for the study and sample size was calculated by taking % change of 24% for the quantity and timing of sugary drinks using the two -proportion comparison (paired) {Mc Nemars test} from study conducted by Hayes *et al.* [18].

## Inclusion criteria:

Healthy children of both genders aged 12 to 71 months (1-6 years), with only primary dentition, whose caregivers were willing to provide consent for the study were included.

## Exclusion criteria:

Children with special healthcare needs, under prolonged antibiotic regimen or other medications which might affect their oral health status or dietary intake, children suffering from any systemic disease and with cleft lip or cleft palate were excluded from study. The Study was conducted after getting approval from the Institutional Ethical Committee (IHEC-LOP/2023/IL0123) and informed written parental consent was obtained before the commencement of the study by explaining the nature of the study. All children aged 1-6 years were examined for the presence of dental caries using decayed, missing and filled teeth (dmft) index. Two dentists and two dental hygienists were trained and supervised in diet assessment and diet counselling. Diet diary was utilized to record sucrose consumption according to Nizel and Papas method. The Dietary Data was collected using structured questions regarding the physical form (liquid, solid) and consistency (sticky, slowly dissolving) of the sugary foods consumed. The "sugar score "based on the frequency and form of consumption of sweets and sugar-sweetened foods were listed and calculated as liquid (frequency multiplied by 5), solid (frequency multiplied by 10) and slowly dissolving (frequency multiplied by 15) [19]. A 24-hr dietary recall was recorded and sweet score was subsequently calculated for recorded diet chart. This sweet score quantifies the overall sugar and serves as an important indicator of an individual's dietary intake, which is significant contributing factor to the development of dental caries [20]. 'Starting The Conversation (STC)' dietary tool questionnaire was used to evaluate the change in dietary practices before and after the intervention, *i.e.* diet counselling of the parents. The post intervention evaluation was done one year after the counselling to assess long term compliance. Clinical management of ECC was provided to all the participants, which included preventive (*i.e.* fluoride application) and curative (*i.e.* restorative and pulp care) procedures.

## Statistical analysis:

Data obtained in the study was analysed using Jamovi software (2.5.6). Proportions were calculated for the categorial variables. Mean and median were calculated for quantitative variables along the standard deviation. Association between pre-counselling and post-counselling STC Score, solid sugar exposure score, liquid sugar exposure score, pre-counselling decayed teeth and post counselling new lesion with age, gender, birth order, parent's education and family type was tested using paired t test and Mann Whitney U test. Value of p < 0.05 was taken as statistically significant.

## Results and Discussion:

The study population consisted of 39 participants, accompanied by their parents. Table 1 (see PDF) summarizes the socio-demographic characteristics of the study population. Participants' age ranged from 1-6 years, with mean±SD of 4.15±0.98 years and majority of them falling within 4-6 year of age group. There was higher proportion of male participants (64.1%) who were affected by ECC with equal distribution across birth order. Most participants' parents had completed their graduation and majority belonged to nuclear families. Table 2 (see PDF) summarizes the descriptive analysis of outcome variables such as pre- and post-counselling STC score, pre- and post- counselling solid sugar exposure score, pre- and post-counselling liquid sugar exposure score, pre- and post-counselling decayed teeth and dmft index. After counselling of the parents there was reduction of solid and liquid sugar exposure which helped in reducing dental caries and shows that diet counselling was effective the mean STC score pre-counselling was 7.82±1.32 which significantly decreased to 5.77±1.27, mean pre-counselling solid sugar exposure score reduced from 15.38±6.43 times/day to 7.95±3.93 times/day, mean pre-counselling liquid sugar exposure score reduced from 8.59±3.23 to 5.26±1.12. Pre-counselling decayed teeth (dt) means teeth that were identified when patient came for initial check-up. After counselling there was significant reduction of decayed teeth, highlighting the positive impact of the counselling and treatment procedure carried out followed by good oral hygiene practice. New decayed lesion means post counselling decayed teeth (dt) after 1 year follow up and this reduced from a mean of 5.36±2.07 to 1.08 ± 0.87. These demonstrate that effective measures taken (diet counselling, good oral hygiene practice, parental guidance to their children) played a successful role in reduction of new carious lesion. The mean initial dmft was 5.87 ± 2.42, which likely represents total number of decayed, missing or filled teeth observed at initial dental visit. Paired t-test showed statistically significant improvements in all the outcome variables after the dietary counselling and treatment protocol (p < 0.001) (Table 3 - see PDF), indicating that the intervention was highly effective. There was also significant reduction in STC, solid sugar, liquid sugar and decayed teeth scores post- counselling (p < 0.001) (Table 3 - see PDF). Table 4 (see PDF) shows association of socio-demographic characteristics with outcome variables. Birth order significantly influenced liquid sugar exposure score both before (p = 0.002) and after counselling (p<0.0001). However, no other significant associations were found between parental education, age, gender and family type with pre- and post-counselling STC scores, solid sugar exposure score, liquid sugar exposure score and decayed teeth.

Dental caries is one of the most prevalent health concerns, affecting the young population worldwide and having significant impact on children leading to difficulty in eating, disturbed sleep, reduced self-esteem and adversely hindering their growth and development [21]. Dental caries is largely preventable with simple cost-effective interventions such as reducing sugar intake since as diet and dental caries has a dose-response relationship (if sugary foods and drinks are consumed more frequently then there is higher risk for development of caries). Dental caries is primarily caused by intake of sugary foods and drinks however malnutrition also plays a significant role. Poor nutrition weakens the tooth enamel and immune system which affects the oral health. So, both diet and nutrition are key factors in preventing dental caries [22]. The present study evaluates the efficacy of dietary counselling in managing ECC and our study findings gave insights that diet counselling has not only improved dietary habits but also promoted healthier behaviours, such as following better oral hygiene practice brushing teeth at night, rinsing the mouth after meals, limiting the sugar intake to meals or snacks and avoiding processed food and drinks and also showed a marked improvement in dietary awareness among parents following counselling [23, 24]. There was significant reduction in both solid sugar exposure and new carious lesions post-counselling which highlights the importance of structured dietary interventions in dental caries prevention. Sugar exposure both in solid and liquid forms recorded through 24 hrs diet diary reduced substantially following diet counselling. Evidence supports that both solid and liquid sugar factors are interrelated to caries [25]. These findings align with previous studies demonstrating that dietary counselling can significantly reduce sugar intake and lowers caries incidence in young children [26]. Studies have shown that parental awareness and engagement in dietary modifications are crucial in reducing the burden of ECC [27]. STC score also improved significantly following diet counselling indicating better dietary practices followed by the caregivers for their children. This improvement agreed with a study conducted by Feldens *et al.* [28] to assess the effectiveness of home visits advising mothers about healthy feeding practices during the first year of life on the occurrence of early childhood caries and severe early childhood caries at 4 years of age. The study concluded that nutritional advice during the first year of life decreases incidence of caries and severity at four years of age in a low-income community. Another study conducted by Chuong *et al.* [29] showed on how children and adolescents with inflammatory bowel diseases (IBD) and their parents coped with the illness by changing their food habits and diet in their daily lives, which helps in its effective management, thereby supporting healthy dietary interventions. Skeie and Klock [30] reported that early intervention will not only prevent progression of caries in the primary dentition but also reduce caries occurrence in the permanent dentition. Another study conducted by Aljafari *et al.* [31] states that intervention should start at an early age and before the onset of dental caries by educating the parents to take daily preventive measures. The present study supports this finding, emphasizing the requirement of amalgamation of dietary interventions and routine paediatric dental care that can have a positive effect on oral health and reduce the risk of tooth decay. Furthermore, our study found a significant association between birth order and post-counselling liquid sugar score (p = 0.0001), suggesting that birth order might influence dietary practices. Prior research has shown that children born later than their sibling often receive less parental attention regarding dietary habits and oral hygiene, leading to increased sugar consumption. Our study adds to this evidence by showing that birth order influences post-counselling dietary behaviours, necessitating targeted interventions for younger siblings in families. Interestingly, no significant associations were found between age, sex, parental education and family type with pre- and post-counselling dietary or caries-related variables. This contradicts some studies suggesting that maternal education and socio-economic status play key roles in dietary choices and caries prevention [32, 33]. The lack of association in our study could be attributed to a relatively homogeneous sample population, thus further research in diverse socio-demographic settings needs to be done. Previous studies have consistently shown that reducing free sugar intake is one of the most effective ways to prevent ECC. Our study supports these findings by demonstrating that counselling effectively reduces sugar exposure and the number of new carious lesions. Moreover, research has suggested that interventions include both dietary counselling and oral hygiene education have a greater impact on caries prevention. Dietary habits can play a crucial role in determining oral health outcomes. In the present study we focussed on counselling of parents to guide them to give preference to natural, homemade food over processed or readymade food since consuming sweetened food and drinks especially in between meals is a significant predictor of dental caries and related to other conditions such as obesity. Counselling sessions can encourage parents to follow balanced diet for their children and therefore not just restrict the sugar intake but more importantly provide adequate nutrition to the child in the form of balanced diet. This holistic approach not only focused on providing proper nutrition and healthy food to children but in turn contributed to decline in dental decay. The dmft index is used worldwide to measure the prevalence of dental caries based on a count of decayed, missing and filled teeth. In the present study initial DMFT score of which majority were decayed teeth during the pre-counselling phase was found to be reduced statistically significantly after the treatment and counselling which shows that adopting healthier dietary habits, limiting free sugar consumption and access to preventive and restorative dental care may decrease caries activity. This finding is similar to other studies on improvement in health parameters seen following diet counselling. A study conducted by Weker [34] evaluated the effectiveness of dietary intervention in treatment of obesity in children lowering fat consumption and making changes to both quantity and quality of food which decreased obesity among children and improved the health markers (lipid profile).

## Conclusion:

Parental counselling on balanced diet, restricting sugar consumption, regular dental visits and proper oral hygiene is essential for preventing dental diseases but this promotes overall growth and development of children. Therefore, diet counselling should be an integral part of routine dental care. As birth order can influence dietary habits, future family-centered approaches should be incorporated into routine paediatric and dental care to improve oral health outcomes for all children.

## Strength and limitations:

Although small sample size (n=39) limits the generability of our findings. Dietary habits were assessed through self-reported measures, which may have recall bias. Secondly, future studies on larger sample sizes, diverse populations and objective dietary assessments (e.g., food diaries, biomarkers of sugar intake) may validate these observations.

## Recommendations:

Healthcare professionals need to emphasize more on awareness programs and provide structured maternal and child healthcare dietary guidance along with clinical care to reduce the burden of ECC.
